# Bacterial contamination of platelet products in the Blood Transfusion Center of Isfahan, Iran

**DOI:** 10.3205/dgkh000283

**Published:** 2016-11-17

**Authors:** Baghi Baghban Farzad, Baghban Farshad, Bamzadeh Zahra, Akbari Nahid, Khosravi Bakhtiari Mahsa

**Affiliations:** 1 Microbiology Department, Shahrekord Branch, Islamic Azad University, Shahrekord, Iran; 2 Microbiology Department, Yasooj Branch, Islamic Azad University, Yasooj, Iran; 3 Blood Transfusion Center of Isfahan, Isfahan, Iran; 4 Clinical Pathology Department, Veterinary Faculty of Shiraz University, Shiraz, Iran

**Keywords:** platelet concentrate, bacterial contamination, PCR

## Abstract

**Aim:** Overall the risk of transfusion transmitted infections has decreased, especially viral infections like HIV and hepatitis B and C. Bacterial contamination of blood and its cellular components, however, remains a common microbiological cause of transfusion associated morbidity and mortality. Platelets pose a special risk given their preservation methods. The incidence of these episodes needs to be assessed and updated on regular basis to accurately manage the risk of transfusion transmitted bacterial infections.

**Method:** 2,000 platelet samples from the Blood Transfusion Center of Isfahan were examined randomly during a 5-month period by bacterial culture and molecular tests. Four platelet samples were found to be contaminated with bacteria, giving a rate of contamination of 500 (0.2%) of tested platelets. Isolated bacteria included one each of *Klebsiella pneumoniae*, *Staphylococcus aureus*, *Staphylococcus epidermidis* and *Staphylococcus haemolyticus*.

**Conclusion:** Our study underlines the need for additional safety procedures like bacterial screening and pathogen reduction technology to further decrease the risk of transfusion associated bacterial infections.

## Zusammenfassung

**Zielsetzung:** Grundsätzlich ist das Risiko transfusionsübertragener Infektionen speziell in Hinblick auf Virusinfektionen wie HIV, HBV und HCV gesunken. Dagegen ist die bakterielle Kontamination von Blut und seiner Komponenten nach wie vor Ursache transfusionsassoziierter Morbidität und Mortalität. Blutplättchen stellen ein besonderes Risiko auf Grund der Herstellung dar. Die Inzidenz derartiger Episoden muss ermittelt werden, um auf dieser Grundlage das Infektionsrisiko zu minimieren.

**Methode:** Es wurden 2.000 Proben von Blutplättchenprodukten des Transfusionszentrums in Isfahan randomisiert im Verlauf von 5 Monaten mikrobiologisch und mittels PCR untersucht. Vier Proben waren kontaminiert (0,2%), davon je eine mit *Klebsiella pneumoniae*, *Staphylococcus aureus*, *Staphylococcus epidermidis* bzw. *Staphylococcus haemolyticus*.

**Schlussfolgerung:** Die Studie unterstreicht die Notwendigkeit für zusätzliche Kontrollmaßnahmen wie bakteriologisches Screening und erregerreduzierende Technologien, um das Risiko transfusionsübertragener Infektionen weiter zu reduzieren.

## Introduction

Bacterial contamination of platelet products occurs at the time of blood collection. Most microorganisms in culture and reported cases of sepsis from contaminated platelet products are natural flora of skin. Because the complete decontamination of human skin is impossible, blood culture results show that after proper skin antisepsis, the rate of positive blood cultures varies from 2 to 6% [1]. The Bacterial infections are the main causes of deaths and diseases related to blood product transfusion. Bacterial contamination of platelet packs is more common than that of red blood cell (RBC) packages, because many of the microorganisms can stay alive and grow under the storage conditions of platelet products (20 to 24°C) but they cannot grow in stored RBC bags (1 to 6°C) [2]. The most common cause of mortality after blood transfusion is infection. The etiologic factors could be viruses, bacteria or protozoa. These organisms can cause clinical disease, remain dormant within the body, or cause asymptomatic infections. All blood banks have screening methods in order to avoid such infections, but most infectious agents cannot be identified due to timeout [3]. Despite optimal collection techniques, low numbers of bacteria commonly enter blood components at the time of phlebotomy. During storage of platelet components bacteria proliferate from relatively low undetectable levels at the beginning of storage time, to relatively high virulent bacteria titers at the end of shelf life. In the cases reported of sepsis associated with platelet transfusions, patients present with fever, hypotension and chills at the time of transfusion or shortly after the transfusion started. Sometimes symptoms can be delayed for 2 weeks after the injection. Half of the patients with shock require pressors to maintain blood pressure. The mortality rate due to injection of platelets infected by bacterial agents is approximately 25%. Most of these reactions occur when the shelf life of platelet products has expired [4]. The Blood Transfusion Center of Isfahan produces about 52,000 units of platelet products a year and approximately 50,000 units are consumed on an annual basis. The purpose of this study is to find the incidence of bacterial contamination of platelet products in the Transfusion Center of Isfahan.

## Materials and methods

This study included the screening of 2,000 samples of platelet products from blood donors collected over a 5-month period. Primary culture and subculture were used to examine these samples. Primary culture included inoculation of the sample into thioglycolate sodium broth (High Media, India). 2 ml of platelet product were added to culture medium and incubated for 5 days at 37°C. After the initial enrichment, samples were examined for turbidity and color change and if there was any change, the samples were subcultured in solid blood agar and eosin methylene blue agar media [5]. Bacterial identification was achieved by phenotypic and genotypic methods as described below.

### Phenotypic identification

To identify the phenotype of isolated bacteria, the macroscopic and microscopic properties of bacteria were used. For macroscopic identification, the color and appearance of colonies were examined. For microscopic study the Gram staining method and conventional biochemical tests in bacteriology were used, such as coagulase test, urease test, citrate test, indole and mannitol test. The API kit (Biomerieux France) was also used for detection of bacteria. Bacterial suspension was added to the small tubes which contained dry chemicals for enzymatic activity and fermentation of carbohydrates and then the tubes were maintained 24 to 48 h at the temperature which was desirable for the organism. After this period, related reagents were added and according to the color change of the reagents the results for each test were evaluated as positive or negative. By the obtained codes and with the help of software the bacteria were identified.

### Genotypic identification

For extraction of DNA, the bacteria were cultured in nutrient agar casein Tripty (Merck, Germany). Identification of isolated bacteria was done by gene amplification 16S rRNA gene of bacteria. At first DNA was extracted by the use of a DNA extraction kit (CinnaGen, Iran) from the samples. The polymerase chain reaction (PCR) was performed using primers F: 5'-CAACGAGCGCAACCCT-3', R: 5'-GGTTACCTTGTTACGACTT-3' corresponding to the gene [6]. To obtain a mastermix with a volume of 25 µl, 18 µl of sterile distilled water, 2.5 µl of buffer at a concentration 10 times, 0.75 µl MgCl_2_, 0.5 µl dNTP, 1 µl forward and reverse primers at a concentration 10 pmol/µl, 0.25 µl DNA polymerase enzyme and 1 µl of template DNA were mixed. Finally, the PCR process was performed by using a thermocycler (Eppendorf, Germany) as follows: the process was adjusted for initial denaturation at 95°C for 180 s, followed by 35 cycles at 95°C for 60 s until secondary denaturation, 56°C for 45 s until fusion and 72°C for 60 s for the elongation phase. For the final elongation at 72°C, a duration of 300 s was considered [6]. The PCR products were electrophoresed on 1% agarose gel. To view the amplified DNA product, the gel was placed under the UV transilluminator and bands were detected along the 50 bP marker.

## Results

In our study we identified 4 infected samples. The infection rate in the present study was 1 in 500 tested platelets (0.02%). The bacteria isolated were *Klebsiella pneumoniae*, *Staphylococcus aureus*, *Staphylococcus epidermidis* and *Staphylococcus haemolyticus*, each in one case.

**Figure 1:**
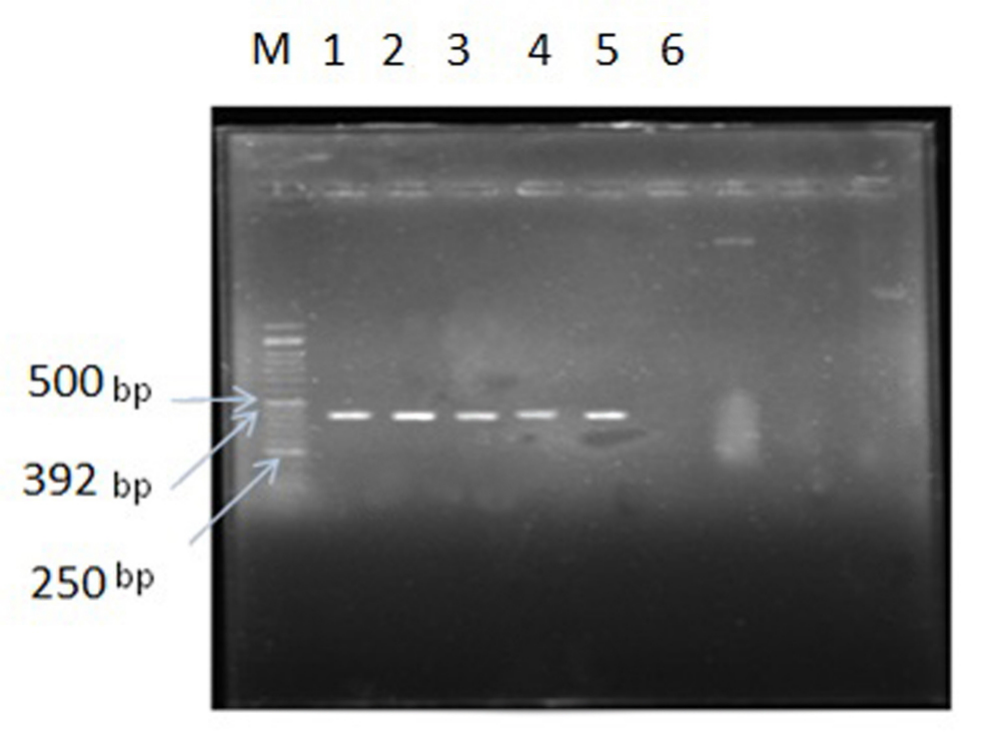
Electrophoresis of PCR products on the 1% agarose gel. M: 50 bp Marker, 1–4: electrophoretic pattern related to the 16S rRNA gene, 5: Positive control, 6: Negative control.

Definitive identification of bacteria was done by the API that confirmed the correct answers. We further used the PCR test for precise identification of isolated bacteria (Figure 1 (Fig. 1)). The PCR products from the four samples were sequenced for the 16SrRNA gene and bacteria were identified at the species level. The sequence results identified case 1 with 98% homology with *Klebsiella pneumoniae*, case 2 with 83% homology with *Staphylococcus aureus* Z172, case 3 with 99% homology with *Staphylococcus epidermidis* RP62A, and case 4 with 90% homology with *Staphylococcus haemolyticus* JCSC1435.

## Discussion

Although the risk of infections transmitted through transfusion is less than in the past, we still come across bacterial infections after transfusion of platelet products. This remains an important cause of morbidity and mortality even in developed countries. Only with continuous improvement and implementation of selected donors, sensitive screening tests and effective inactivation methods the risk of bacterial infections transmitted through transfusion can be reduced. According to the report of the Red Cross of America, Gram-positive aerobic bacteria are the most common pathogenic isolates (75%) from platelet products [2]. Platelet products can be stored at room temperature for more than 5 days and this provides the optimal conditions for the growth of bacteria. Sources of bacterial contamination of platelet products could be donor bacteremia or contamination from the surface of the skin during injection into the vessel. The latter is more prevalent because the microorganisms recovered from contaminated platelets are part of the skin flora. Deeper layers of the skin, hair follicles and sebaceous glands carry bacteria which cannot be removed even after antisepsis by mechanical methods. When platelet concentrates are stored, the temperature and constant agitation support bacterial proliferation from low undetectable levels to relatively high bacterial titers and endotoxin generation at the end of shelf life [7]. Aerobic bacterial culture studies show that 1 in 1,000 to 2,000 platelet units are usually found to be infected [8]. In America after transfusion mistakes, bacterial contamination is the second most prevalent cause of death related to blood transfusion. In America the estimated number of patients who received infected platelet products was 2,000 to 4,000, which resulted in 20 to 600 cases of clinical sepsis and in 40 to 533 deaths. The mortality rate in America is estimated to be about one death per 500,000 units of platelets [9]. From 1995 to 2001, 21 cases of bacterial contamination were reported by the blood surveillance system of England, 6 of them leading to death, of which 5 cases were related to the contamination of platelet products. In France the rate of transfusion reactions due to bacterial contamination of platelet products has been reported to be about 1 in 25,000 platelet units as well [9]. In Qatar, in 1997, a total of 200 platelet samples were analyzed for bacterial growth. On the first day, 3–5 h after blood collection, the samples were tested. The same platelet bags were tested again after the expiration date (fifth day). The frequencies of positive cultures in the early days were not significantly higher than on the fifth day. Most isolated organisms were from the skin flora. In this study, 16 organisms were isolated, of which 15 were aerobic organisms and 1 was an anaerobic organism [10]. Hillyer et al. [11], studying the risk of bacterial contamination of blood components, found that isolated organisms from contaminated platelet units were mostly from the skin flora and Gram-positive cocci. Brecher and Hay [8] declared that Gram-negative organisms were the main reason for the deaths due to transfusion of infected blood products (59.7%). Their study showed that Gram-positive organisms such as *Staphylococcus epidermidis* and *Bacillus cereus* were present in 41 of 58 cases (71%), but Gram-negative organisms (mostly Enterobacteriaceae) caused the highest mortality rate of 9 of 11 deaths. Adjei et al. [12] examined 330 samples of whole blood, plasma and platelets from different locations in Ghana for bacterial contamination. From 22 platelet product samples, only 2 samples (9%) were positive. In this study, the incidence of bacterial contamination of blood products was determined to be 9% (28 cases of 303 samples). Furthermore, in this study, the rate of whole blood contamination was determined to be 13% (24 cases of 192 samples), and the rate of plasma contamination was determined to be 3% (12 cases of 79 samples). The Gram-positive isolates in this study were coagulase-negative *Staphylococcus*, *Staphylococcus aureus*, *Bacillus* spp., and the Gram-negative bacteria isolated were *Yersinia enterocolitica*, *Citrobacter freundii*, *Escherichia coli*, *Pseudomonas aeruginosa* and *Klebsiella pneumoniae*. Bolarinwa et al. [13] studied bacterial contamination of blood components in three hospitals in Nigeria and found that all contaminated samples were whole blood; the isolated bacteria were Gram-positive bacteria including *Staphylococcus aureus*, coagulase negative *Staphylococcus*, *Bacillus* spp. and *Listeria* spp. In this study of 162 blood products, 160 samples of compact cells and whole blood and 2 platelet products were examined; 14 samples (8.8%) were determined to be infected. Ahmadi et al. [5] examined the bacterial contamination of platelet concentrates prepared in the Tehran Blood Transfusion Center and isolated *Staphylococcus epidermidis* (4 cases), *Staphylococcus saprophyticus* (2 cases), *Acinetobacter* spp. (5 cases), and *Bacillus* spp. (3 cases) [5]. Benjamin et al. [14] examined bacterial culture of platelet products free from antibodies and found the same bacterial species that are associated with sepsis [14]. 

In conclusion, transfusion-related bacterial infections remain the significant cause of morbidity and mortality. Our study identified at least 4 cases of contaminated platelet packs which could potentially have caused serious complications. We urgently need technology for reduction of pathogens such as viruses, fungi, bacteria, parasites and their inactivation as a way to prevent their transmission through injection of blood products.

## Notes

### Competing interests

The authors declare that they have no competing interests.

### Acknowledgements

The authors would like to thank Mr. Mostafa Aghahosseini, the assistant of the Blood Transfusion Center of Isfahan, and Dr. Fakhrolmolouk Yavari, Mr. Saeid Rezaei, Mr. Farzin Nasiri, the Blood Bank personnel, Dr. Mino Adib, the Chief of Sepahan Hospital Laboratory, and Mr. Mansour Baghban, the Laboratory Expert of Sepahan Hospital, for their assistance with completing this project.

## References

[1] Hillyer CD, Josephson CD, Blajchman MA, Vostal JG, Epstein JS, Goodman JL (2003). Bacterial contamination of blood components: risks, strategies, and regulation: joint ASH and AABB educational session in transfusion medicine. Hematology Am Soc Hematol Educ Program.

[2] Bihl F, Castelli D, Marincola F, Dodd RY, Brander C (2007). Transfusion-transmitted infections. J Transl Med.

[3] Abrol P, Lal H, Puneet Kaur Kochhar (2012). Transfusion-Transmitted Bacterial, Viral and Protozoal Infections. Blood Transfusion in Clinical Practice.

[4] Razjoo F, Dabirmoghdan A (2011). Bacteria and Transfusion.

[5] Ahmadi J, Gholizadeh HR, Farseh R, Sharifi S (2006). Evaluation of bacterial contamination of platelet concentrates collected at Tehran Regional Blood Center. Sci J Iran Blood Transfus Organ.

[6] Bamzadeh Z, Baserisalehi M, Bahador N, Hejazi SH (2013). Screening of soil Streptomyces and characterization of their bioactive compounds. Health Med.

[7] (2003). Detection of bacterial contamination in platelet components. Blood Bull.

[8] Brecher ME, Hay SN (2005). Bacterial contamination of blood components. Clin Microbiol Rev.

[9] Védy D, Robert D, Gasparini D, Canellini G, Waldvogel S, Tissot JD (2009). Bacterial contamination of platelet concentrates: pathogen detection and inactivation methods. Hematol Rev.

[10] Ahmed F, Elhag K (1997). Bacterial contamination of platelet concentrate units: a study on 200 units. Qatar Med J.

[11] Hillyer CD, Lankford KV, Roback JD, Gillespie TW, Silberstein LE (1999). Transfusion of the HIV-seropositive patient: immunomodulation, viral reactivation, and limiting exposure to EBV (HHV-4), CMV (HHV-5), and HHV-6, 7, and 8. Transfus Med Rev.

[12] Adjei AA, Kuma GK, Tettey Y, Ayeh-Kumi PF, Opintan J, Apeagyei F, Ankrah JO, Adiku TK, Narter-Olaga EG (2009). Bacterial contamination of blood and blood components in three major blood transfusion centers, Accra, Ghana. Jpn J Infect Dis.

[13] Bolarinwa RA, Aboderin OA, Odetoyin BW, Adegunloye AB (2011). Bacterial contamination of blood and blood components in a tertiary hospital setting in Nigeria. Int J Infect Control.

[14] Benjamin RJ, Dy B, Perez J, Eder AF, Wagner SJ (2014). Bacterial culture of apheresis platelets: a mathematical model of the residual rate of contamination based on unconfirmed positive results. Vox Sang.

